# Implementation of k_L_a-Based Strategy for Scaling Up *Porphyridium purpureum* (Red Marine Microalga) to Produce High-Value Phycoerythrin, Fatty Acids, and Proteins

**DOI:** 10.3390/md19060290

**Published:** 2021-05-21

**Authors:** Laura Isabel Rodas-Zuluaga, Carlos Castillo-Zacarías, Gabriela Núñez-Goitia, María Adriana Martínez-Prado, José Rodríguez-Rodríguez, Itzel Y. López-Pacheco, Juan Eduardo Sosa-Hernández, Hafiz M. N. Iqbal, Roberto Parra-Saldívar

**Affiliations:** 1 School of Engineering and Sciences, Tecnologico de Monterrey, Monterrey 64849, Mexico; laura.rodas@tec.mx (L.I.R.-Z.); carlos.castillozcr@uanl.edu.mx (C.C.-Z.); jrr@tec.mx (J.R.-R.); yolotzinlopez@tec.mx (I.Y.L.-P.); eduardo.sosa@tec.mx (J.E.S.-H.); 2 Chemical & Biochemical Engineering Department, Tecnológico Nacional de México-Instituto Tecnológico de Durango, Blvd. Felipe Pescador 1830 Ote. Durango, Durango 34080, Mexico; 14041243@itdurango.edu.mx (G.N.-G.); adriana.martinezprado@itdurango.edu.mx (M.A.M.-P.)

**Keywords:** *Porphyridium purpureum*, scaling-up process, volumetric mass transfer coefficients, phycoerythrin, high-value metabolites

## Abstract

*Porphyridium purpureum* is a well-known Rhodophyta that recently has attracted enormous attention because of its capacity to produce many high-value metabolites such as the pigment phycoerythrin and several high-value fatty acids. Phycoerythrin is a fluorescent red protein-pigment commercially relevant with antioxidant, antimicrobial activity, and fluorescent properties. The volumetric mass transfer coefficient (k_L_a) was kept constant within the different scaling-up stages in the present study. This scaling-up strategy was sought to maintain phycoerythrin production and other high-value metabolites by *Porphyridium purpureum*, using hanging-bag photobioreactors. The k_L_a was monitored to ensure the appropriate mixing and CO_2_ diffusion in the entire culture during the scaling process (16, 80, and 400 L). Then, biomass concentration, proteins, fatty acids, carbohydrates, and phycoerythrin were determined in each step of the scaling-up process. The k_L_a at 16 L reached a level of 0.0052 s^−1^, while at 80 L, a value of 0.0024 s^−1^ was achieved. This work result indicated that at 400 L, 1.22 g L^−1^ of biomass was obtained, and total carbohydrates (117.24 mg L^−1^), proteins (240.63 mg L^−1^), and lipids (17.75% DW) were accumulated. Regarding fatty acids production, 46.03% palmitic, 8.03% linoleic, 22.67% arachidonic, and 2.55% eicosapentaenoic acid were identified, principally. The phycoerythrin production was 20.88 mg L^−1^ with a purity of 2.75, making it viable for food-related applications. The results of these experiments provide insight into the high-scale production of phycoerythrin via the cultivation of *P. purpureum* in an inexpensive and straightforward culture system.

## 1. Introduction

*Porphyridium purpureum* is a unicellular marine microalga from the phylum Rhodophyta with spherical-shaped cells of about 8–15 μm in size. This microalga performs photosynthesis by the lamella-shaped thylakoids that host the photosynthetic material such as phycobiliproteins (phycoerythrin, phycocyanin, and allophycocyanin) and chlorophyll. This latter is located inside the thylakoids, and the phycobiliproteins are organized in a hemispherical structure called phycobilisome located outside of the thylakoid membrane [1,2]. *P. purpureum* is an invaluable source of several natural active compounds. For instance, phycoerythrin is the major phycobiliprotein present in the phycobilisome, and it is responsible for the pink-reddish color in *P. purpureum*. Phycoerythrin application in biomedicine has been widely studied, specifically in flow cytometry and microscopy. This protein is highly fluorescent with an absorption coefficient and quantum yield superior to other fluorescent proteins [3]. Its use in the food and cosmetic industry as a natural dye has been investigated [2,4,5,6]. According to some researchers, the cost of phycoerythrin is around 50 USD per milligram [7]. This factor and the growing demand for natural dyes make attractive the idea of developing efficient processes for its production and purification [8]. In addition to phycoerythrin, *P. purpureum* produces multiple high-value metabolites such as extracellular polysaccharides and several polyunsaturated fatty acids, mainly arachidonic (C20:4n6) and eicosapentaenoic acids (C20:5n3) [1,9,10]. Different types of technology have been developed for large-scale cultivation to improve the industrial exploitation of the metabolites obtained from *P. purpureum*. This microalga has grown either in open culture systems (open ponds) or in closed systems, such as a bubble column, serpentine tube, annular column, and flat plate [2]. The closed system has been shown to have higher yields; for example, Cohen and Arad [9] obtained almost 300% more *Porphyridium* sp. biomass using a closed culture system.

**Figure 1:**
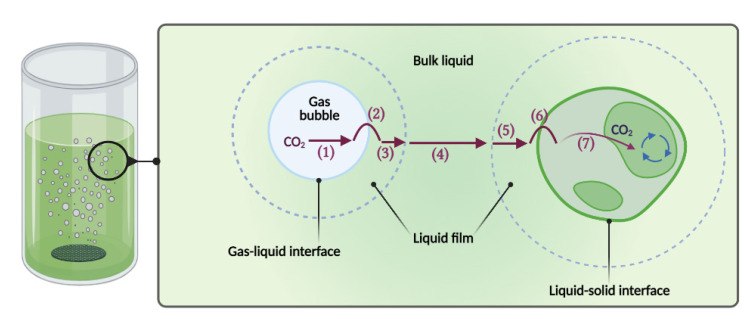
Steps for CO_2_ transfer from gas bubble to cell. (**1**) Transfer from the interior of the bubble to the gas–liquid interface. (**2**) Movement across the gas–liquid interface. (**3**) Diffusion through relatively stagnant film surrounding the bubble. (**4**) Transport through the bulk liquid. (**5**) Diffusion through the relatively thick film surrounding the microalgae. (**6**) Transport into the microalgae. (**7**) Transport through the cytoplasm to the site of the reaction. The Figure was created with BioRender.com template and exported under the terms of premium subscription.

However, scaling-up microalgae growth is sometimes an expensive process that must be performed strategically to maintain the laboratory scale’s yields. Scaling-up photobioreactors can be done by increasing the length, diameter, height, number of units in the culture systems (known as a numbering-up process), and by maintaining a constant geometric similarity, Reynolds number, agitation power per reactor volume, the shear stress level, and the mixing time. These scale-up strategies are challenging because maintaining optimum light, temperature, mixing, and mass transfer in photobioreactors at large volumes is difficult [10,11,12,13,14]. The overall mass transfer coefficient (k_L_a) is the most commonly used parameter for evaluating photobioreactor performance and improving microalgae productivity [11,15,16,17,18]. Commonly, the k_L_a has been used as a scaling-up parameter in heterotrophic culture systems (i.e., bacteria and yeasts). Similarly, the k_L_a can be an excellent tool for the photobioreactors’ scaling-up process [19,20]. This parameter determines the transfer rate of CO_2_ from the gas phase to the liquid phase. As can be seen in Figure 1, this process involves three mass transfer steps: gas (CO_2_)–liquid (medium), gas (CO_2_)–solid (microalgae), and liquid (medium)–solid (microalgae) [21,22]. Since CO_2_ is the primary carbon source of microalgae, the transfer of this gas is decisive for its growth and subsequent production of high-value metabolites [11]. However, some large-scale photobioreactors with acceptable *Porphyridium* spp. biomass productivities have been developed using plastic (polyethylene) bag systems which are cheaper than conventional locked photobioreactors [2,9,23,24]. In addition, high-value metabolites have been produced from microalgae using different materials and methods [25,26].

Nevertheless, the biochemical composition of *Porphyridium* spp. and the potential for the co-production of multiple products based on a biorefinery approach have never been fully implemented and remain a challenge because of the cost of the overall process [27]. Therefore, this study’s objective was to scale up the culture of *Porphyridium purpureum*, using the k_L_a as a scale parameter in a low-cost hanging-bag photobioreactor system. Additionally, the co-production of phycoerythrin and other high-value metabolites of interest, such as carbohydrates, proteins, lipids, and polyunsaturated fatty acids, was evaluated.

## 2. Result and Discussion

### 2.1. Volumetric Mass Transfer Coefficients

Photosynthesis is a complex process through microalgae that utilize solar energy and several essential nutrients (C, N, P, S, K, Fe, etc.) to synthesize their biomass compounds and multiply their cells [28]. *Porphyridium purpureum* is an autotrophic microorganism that uses CO_2_ to grow and metabolize inorganic carbon sources [29]. For efficient CO_2_ uptake, the incoming gas phase with CO_2_ should be transferred to the aqueous phase, making it available to grow the microalgae in the photobioreactor. The volumetric mass transfer coefficient (k_L_a) determines the transfer rate of CO_2_ from the gas phase to the liquid phase. As previously reported, an efficient mass transfer has essential effects on photosynthetic efficiency, productivity, and cell composition [30]. To scale up the *P. purpureum* culture while maintaining the biomass and metabolites productivity, the k_L_a was determined in the 16 L culture, operated under standard cell-free conditions: using F/2 medium at pH 9, 20 °C, and with an airflow of 25 L min^−1^, obtaining a k_L_a value of 0.0052 s^−1^. Then, the k_L_a was determined in the 80 L plastic bag, operated under the same conditions mentioned for the 16 L culture, but with different airflows (50, 70, and 80 L min^−1^). The main reason is that the solubility of CO_2_ in aquatic environments varies significantly and depends on pH, salinity, pressure, and temperature, i.e., CO_2_ solubility in water decreases with decreasing pH [28,31].

On the other hand, CO_2_ solubility rises when pressure increases, affecting the k_L_a [28]. The k_L_a values obtained in the 80 L photobioreactor, using airflows of 50, 70, and 80 L min^−1^, were 0.0007, 0.0022, and 0.0024 s^−1^, respectively. The effect that different airflow rates had on the volumetric mass transfer coefficient is shown in Table 1. It can be noticed that the increase in the airflow rate improved the k_L_a, as other authors have shown [16].

Additionally, the amount of air supplied improved mixing of the medium, keeping algal cells in suspension, eliminating thermal stratification, helping nutrient distribution, improving gas exchange, reducing the degree of shading, and the probability of photoinhibition. However, it has been reported that an excessive increase in the airflow in scaled-up cultivation of microalgae can cause shear stress, damaging the cell and affecting its growth and the production of metabolites of interest [13,15,23]. The highest airflow (80 L min^−1^) was used for the culture of *P. purpureum* in the 80 L plastic bags and for the five individual plastic hanging-bag units that formed the 400 L culture.

**Figure 2:**
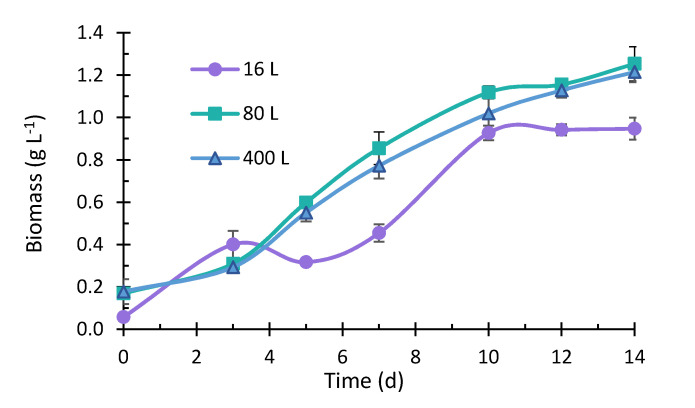
Microalgae *Porphyridium purpureum* growth in 16 L, 80 L (±SD, *n* = 3), and 400 L (±SD, *n* = 5).

**Figure 3:**
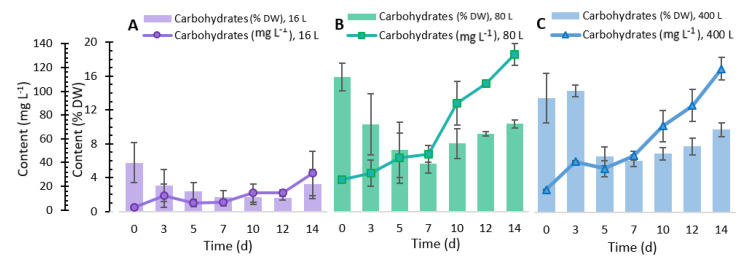
Total carbohydrate content in *Porphyridium purpureum* in the different cultures. (**A**) 16 L, (**B**) 80 L, and (**C**) 400 L. ±SD, *n* = 3.

**Figure 4:**
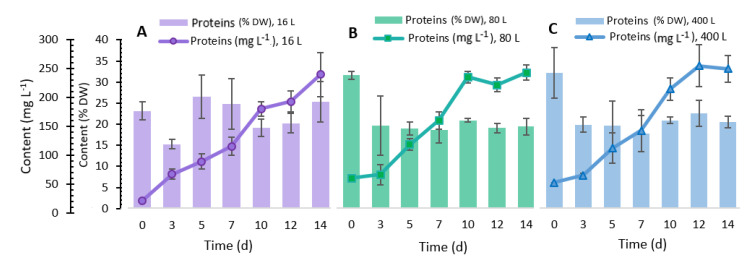
Total protein content in *Porphyridium purpureum* in the different cultures (**A**) 16 L, (**B**) 80 L, and (**C**) 400 L. ±SD, *n* = 3.

The highest k_L_a values obtained in this study (0.0024 s^−1^ and 0.0052 s^−1^) agreed with those determined by Merchuk et al. [24], where *Porphyridium* sp. was cultivated in different reactors (bubble column, airlift, and airlift with a helical flow promoter), achieving a k_L_a between 0.0017 and 0.0047 s^−1^. Furthermore, Rebollo Fuentes et al. [32] reported a k_L_a of 0.0023 s^−1^ for the *Porphyridium cruentum* culture. Other studies have reported k_L_a values between 0.002 and 0.020 s^−1^ in microalgae grown in bioreactors of different geometries [11]. Additionally, maintaining the k_L_a in the scaling process in the present study allowed us to keep the yields of biomass production in the same range (Figure 2), carbohydrates (Figure 3), and proteins (Figure 4). Other studies where microalgae cultivation has been studied and used k_L_a as a scaling parameter managed to maintain lipid production yields such as docosahexaenoic acid from *Schizochytrium* sp. [33,34] and carotenoids by *Haematococcus pluvialis* [35].

### 2.2. Microalgae Growth

The biomass production from the culture of *P. purpureum* under different volumes is presented in Figure 2. The growth curves corresponding to each volume showed that microalgal cells immediately adapted to the medium. Only in the 16 L culture, a drop in the microalgae’s growth was observed since the biomass produced ranged from 0.40 ± 0.064 g L^−1^ to 0.32 ± 0.012 g L^−1^, from days 3 to 5. After that, an increase in growth was observed, and the biomass reached 0.93 ± 0.035 g L^−1^ on the 10th day. Between days 10 and 14 of cultivation, there was no significant growth in this culture, and the highest biomass content was 0.95 ± 0.052 g L^−1^, reached on the 14th day. The 80 and 400 L cultures, performed in plastic bags, showed a similar growth curve and reached 1.25 ± 0.081 g L^−1^ and 1.22 ± 0.049 g L^−1^, respectively, higher than the biomass concentration obtained for the 16 L culture. Assunção et al. [36] scaled up *P. purpureum* from 200 mL to 10 L, obtaining higher yields on a larger scale (0.54 g L^−1^ and 0.73 g L^−1^, respectively) [18,37]. Moreover, the improved cell biomass concentration at higher volumes could be produced when passing an adapted and healthy physiological state inoculum to the following cultures (from 16 to 80 L and 80 to 400 L). Other authors have obtained biomass production yields of 0.95 g L^−1^ [38] and 1.22 g L^−1^ [39] in *P. purpureum* cultures at laboratory scale, and 0.96 g L^−1^ [40], and 1.3 g L^−1^ [41] in 600 and 175 L cultures (using plastic bags as a bioreactor). These results were similar to those obtained in this study, so this method whereby the volume is increased using repeated units of the cultivation system is a viable option to obtain good biomass productivity. The final biomass per liter achieved by the 80 and 400 L cultures was higher than the 16 L culture.

In contrast, the biomass growth rate was higher for 16 L than for 80 and 400 L cultures, and this can be seen in Figure 2 by a more significant slope in two sections of the biomass accumulation trend from the initial inoculum to day 3 and from days 7 to 10; the growth rate is presented in Table 2. Furthermore, according to the statistical analysis performed, no significant differences in the biomass production in 80 and 400 L cultures were found on the 14th, thus indicating that the scaling strategy used allowed maintaining the production of biomass. In the case of doubling time, these values increased when the volume of the culture incremented. Since no significant differences were found concerning the biomass production in the five bags that formed 400 L, the determination of carbohydrates, lipids, proteins, and phycoerythrin was performed in three bags taken as a representative sample of the culture.

### 2.3. Total Carbohydrate Content

Carbohydrates produced by microalgae act as structural components in the cell walls and storage components inside the cell [42]. The cultivation of *P. purpureum* at the different scaling volumes was performed to measure total carbohydrates produced. Figure 3 shows the different production profiles achieved during the 14 days of experimentation. The 16 L volume cultures showed a low production of total carbohydrates, with a maximum production concentration of 32.10 mg L^−1^. Regarding the 80 L culture, this presented a stable and ascending profile of total carbohydrate content, from the 1st day of incubation until the 14^th^ day, with a maximum of 128.14 mg L^−1^. The 400 L culture showed a production profile similar to that shown by the 80 L culture. On day 14, the 400 L culture generated a concentration of around 117.24 mg L^−1^. The results obtained in the 80 and 400 L cultures were consistent with the findings of the study by Razaghi et al. [39], who reported a total carbohydrate content of 130 mg L^−1^ in the culture of *P. cruentum* with F/2 medium at similar nitrate and phosphate concentration, light intensity, and temperature.

In the three scaled-up volumes, the carbohydrate fraction showed a decrease from days 0 to 7. A slight increase occurred from days 7 to 14, obtaining values in the range of 2 to 3% (16 L), 8 to 10% (80 L), and 7 to 9.4% (400 L). The sharp increase in the percentage of carbohydrates in the last eight days of culture may be related to *P. purpureum*; the cell growth caused a decrease in the concentration of nitrogen in the medium, and the reduction in this nutrient trended the accumulation of carbohydrates [27,43]. A study performed by Schulze et al. [44] showed some of the monosaccharides found in *P. purpureum*. These single sugars were galactose, glucose, mannose, arabinose, ribose, and xylose. However, the percentage of carbohydrates found in this study was lower than that reported by other authors who grew *Porphyridium* under similar conditions [45]. From the statistical analysis, we determined that no significant differences were found in the carbohydrates production in 80 and 400 L cultures on the 14th day, which is attributed to the maintenance of k_L_a. 

### 2.4. Lipid Analysis

The analysis of total lipids produced by *P. purpureum* was performed on the 14th cultivation day. In the 16 L culture, 23.59 ± 2.77% of lipids were obtained. In contrast, the 80 L culture had a 15.16 ± 0.79% lipid production. Regarding the 400 L culture, production of 17.75 ± 2.58% lipids was achieved. According to the statistical analysis, we found significant differences between the lipids obtained in the three different volumes under study (16, 80, and 400 L). Similar concentrations of total lipids have been reported in other studies; Oh et al. [45] obtained between 2.2 and 19.3%, and Coward et al. [46] obtained between 19.6 and 21.78%, using different culture conditions. Our results together indicated that a lower light irradiance in the 16 L photobioreactor produced this difference in the lipid content due to its size and shape. This observation is consistent with previous studies that increasing light intensity reduced lipid content [47]. Nevertheless, strains respond differently to photo stress, as suggested by Brindhadevi et al. [48].

Table 3 displays the total lipid and the fatty acid profile of *P. purpureum* cultivated under different volumes regarding lipid production. Palmitic acid was the fatty acid present in the highest proportion (40.41± 3.92, 44.46 ± 4.12, and 46.03 ± 3.89% in 16, 80, and 400 L, respectively). This is a profile usually obtained by other studies; for instance, some have shown a higher concentration of palmitic acid than that obtained in the present study [9,27,49]. Total fatty acid (TFA) contents reported in red microalgae such as *Porphyridium* spp. are usually low; however, the production of fatty acids such as arachidonic and eicosapentaenoic in *P. purpureum* and *P. cruentum* was evaluated. C20:4n6 and C20:5n3 play an important role in the secretion of prostaglandin, thromboxane, and leukotriene, promoting infants’ brain development [49,50]. Although C20:4n6 (between 22.34 and 32.57%) and C20:5n3 (2.50 and 6.14%) were found in this study, these values were lower than those reported by other authors [43,49]. Caprylic [51], stearic, oleic, and linoleic acid [27,46] have also been obtained from *Porphyridium* sp. by other authors.

### 2.5. Total Protein Content

Regarding protein production, the culture of *P. purpureum* showed an interesting profile, observed in Figure 4. It can be seen that for the low-volume culture (16 L), protein production increased throughout the 14 days of incubation. However, compared to the 80 and 400 L cultures, this protein production was lower during the first 13 days of incubation. In the 80 L system, this culture showed a constant and increasing production during the first 10 days of incubation. Subsequently, in the last four days, a stationary production phase occurred. The 400 L system also showed a continuous and upward production during the first 12 days of incubation; however, a slight decrease occurred in the last two days of incubation. These 16, 80, and 400 L culture systems showed a protein production of 231.62 ± 38.151, 240.91 ± 13.234, and 240.63 ± 23.396 mg L^−1^, respectively, at 14 days of incubation. We found no significant difference in protein production in 80 and 400 L cultures from the statistical analysis, highlighting that these results were higher than those obtained in 16 L culture. However, other authors have reported higher protein production from the cultivation of *P. purpureum* (from 0.30 to 1.01 g L^−1^) using another source of nitrogen, since the production of proteins in these microalgae is linked to environmental and culture conditions, especially to concentration and source of nitrogen in the culture media [49].

Concerning the protein percentage content in *P. purpureum*, the culture of 16 L showed values from 14.80 ± 1.0 to 24.57 ± 4.6% DW, reaching the highest value on the 14th day. In 80 and 400 L cultures performed in the plastic bags, the highest values were exhibited at the beginning of the culture (31.48 ± 0.98 and 31.15 ± 5.80%, respectively). During the rest of the experiment, the values were maintained between 18.44 ± 3.08 to 20.97 ± 0.41% for the 80 L and 17.17 ± 4.14 to 21.88 ± 2.95% in the 400 L culture. In the study reported by Fuentes-Grünewald et al. [40], a higher amount of protein (approximately 22%) was obtained in an early stage of the culture of *P. purpureum*. This value decreased to about 15%, similar to the value obtained in the present research. In other *Porphyridium* species the amino acid profile has been studied [52,53], and a high content of essential and non-essential amino acids was found. Some of the amino acids found in *P. cruentum*, a strain related to *P. purpureum*, were aspartic acid, threonine, serine, glutamic acid, glycine, alanine, cysteine, valine, methionine, isoleucine, leucine, phenylalanine, histidine, lysine, arginine, tryptophan, ornithine, and proline. Compared to other food proteins, this amino acid profile makes these microalgae an alternative human food protein source [53].

### 2.6. Phycoerythrin Content

**Figure 5:**
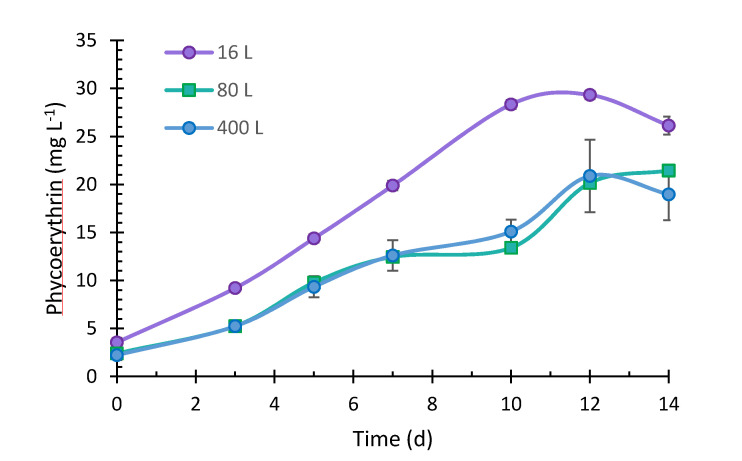
Phycoerythrin production of *Porphyridium purpureum* in 16 L, 80 L, and 400 L (±SD, *n* = 3).

The kinetics of phycoerythrin production by *P. purpureum* is presented in Figure 5. Phycoerythrin increased rapidly from day 0 to day 12, and phycoerythrin accumulation gradually decreased after 12 days. The maximum phycoerythrin content (3.11 ± 0.12% DW) in the 16 L culture was reached at day 12, with a production of 29.32 ± 0.42 mg L^−1^. In the 80 L culture, the phycoerythrin content between days 1 and 12 was equivalent to the 400 L culture. The 80 L production ranged from 2.40 ± 0.07 to 21.11 ± 0.50 mg L^−1^ during the 14 days of culture, and 2.23 ± 0.50 to 20.88 ± 2.69 mg L^−1^ in 400 L culture, from days 0 to 12. Likewise, the higher phycoerythrin contents in 80 and 400 L were 1.74 and 1.85%, respectively. We found a significant difference in the three culture systems’ phycoerythrin production (16, 80, and 400 L) from the statistical analysis. Noticeably, phycoerythrin production was higher in the 16 L; the 16 L culture container had a smaller diameter than the plastic bags, which allowed a greater incidence of light to the culture [54]. Sosa-Hernández et al. [55] correlated higher phycoerythrin production with increased light intensity. Moreover, it is essential to highlight that metabolite production may decrease when a process is scaled at a higher volume because it is difficult to control the variables that affect growth and, therefore, the phycoerythrin production [27,29]. However, in the present study, similar values were obtained to those reported by Marcati et al. [56] (15 mg L^−1^) and Sosa-Hernández et al. [55] (14.66 mg L^−1^), with *P. cruentum* and *P. purpureum*, respectively. Besides, Guihéneuf and Stengel [27] reported a 33.3 mg L^−1^ production, equivalent to 1.96% DW, similar to the percentage of phycoerythrin obtained in our study in the 80 L (1.74%) and 400 L (1.85%) cultures. On the other hand, after filtration through 10 and 2 kDa membranes, a purity of 2.75 was obtained, similar to that reported by Marcati et al. [56], using a similar purification system. The advantage of using this purification method is to treat high volumes of samples quite rapidly, obtaining a phycoerythrin purity index classified as food-grade [57]. This pigment is used as fluorescent markers in biomedical research. It also has applicability in medical and cosmetic industries as a natural colorant and active compound since it has antioxidant [58], antimicrobial, and anticancer activity [57]. However, the extract produced in this study showed excellent potential as an additive and functional ingredient of food due to its nature, unique color, and biological activity because of its food-grade status.

## 3. Material and Methods

### 3.1. Microalgal Strain and Culture Conditions

The scaling-up study of *Porphyridium purpureum* UTEX LB275 (UTEX Culture Collection of Algae, Austin, TX, USA) was performed following the growth conditions optimized previously [55]. The microalgae were grown at 20 °C, illuminated under white light with an intensity of 100 µmol m^−2^ s^−1^ (InstantFit TB LED, PHILIP, La Herradura, México), and the inoculum medium ratio was 2:8 *v*/*v*. *P. purpureum* was cultured in a 20 L polycarbonate carboy, with a 26 cm diameter and 50 cm long, and 16 L of culturing medium. This entire medium was used to inoculate a plastic bag, 40 cm wide and 200 cm long (S-2942, Uline, Apodaca, Mexico), with a working volume of 80 L. This plastic bag was used to inoculate five plastic bags to obtain a final volume of 400 L of culture. The 400 L culture system was built increasing the number of the 80 L culture system units and applying a numbering-up process [14,59,60,61].

**Figure 6:**
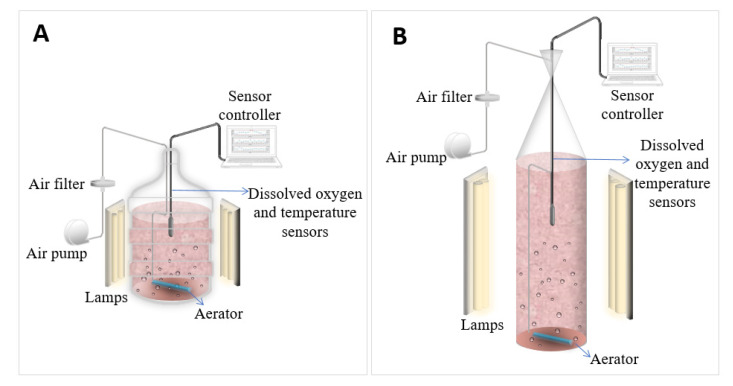
Schematic of the configuration set-up. (**A**) 16 L, (**B**) 80 L.

A schematic representation of the experimental set-up is shown in Figure 6. The culture medium used was f/2 modified with macronutrients (NaNO_3_ 880 µM, NaH_2_PO_4_·H_2_O 36 µM, and Na_2_Si·9H_2_O 106 µM), a trace metal solution (ZnSO_4_·7H_2_O 0.08 µM, MnSO_4_·H_2_O 0.9 µM, Na_2_MoO_4_·2H_2_O 0.03 µM, CoSO_4_·7H_2_O 0.05 µM, CuCl_2_·2H_2_O 0.04 µM, Fe(NH_4_)_2_(SO_4_)·6H_2_O 11.7 µM, and Na_2_EDTA·2H_2_O 11.7 µM), and three vitamin solutions (cyanocobalamin 0.1 mM, biotin 0.1 mM, and thiamine-HCl 1 mM) in HEPES buffer 50 µM. The macronutrient solution was autoclaved (SQ500, Yamato Scientific Co. Ltd., Shanghai, China) at 121 °C for 15 min. The trace metal and vitamin solutions were sterilized using a 0.22 µm filter (431097, CORNING, Reynosa, Mexico). In all the scaling stages, the cultures were aerated with an air pump (ACO-009E, HAILEA, Guangzhou, China) connected to a 0.22 µm air filter (Aervent 50, Merck, Juárez, Mexico), and the air was injected through aerating stones (A972, Marina, Radnor, PA, USA), providing the airflow necessary to reach the k_L_a desired.

### 3.2. Real-Time Oxygen and Temperature Monitoring

A LabView (National Instruments, Munich, Germany) data acquisition program was developed to obtain real-time oxygen and temperature monitoring. An oxygen probe with a response time of 1 s (Atlas Scientific, #ENV-40-DOX, Long Island City, New York, NY, USA) and a temperature probe (RDO PRO-X Probe, In Situ, Fort Collins, CO, USA) were used. Furthermore, temperature and oxygen measurements were registered directly in a spreadsheet (Microsoft Excel, Redmond, WA, USA).

### 3.3. Volumetric Mass Transfer Coefficients

The volumetric mass transfer coefficient (k_L_a) was selected as a scale-up parameter and determined in a free-cell medium by the dynamic absorption method. Briefly, to estimate the k_L_a(*O*_2_), the oxygen was eliminated in the liquid phase by bubbling nitrogen until the oxygen concentration was equal to zero. Then, the liquid was set in contact with air, measuring the oxygen concentration increase (Atlas Scientific, Long Island City, New York, NY, USA) until saturation. The dissolved oxygen concentration variation with time (t) was obtained, and k_L_a(*O*_2_) was calculated according to the following equation:

(1) $$ln(C^{*}{}_{O_{2}}-C_{O_{2}}\left(t\right))=ln\left(C^{*}{}_{O_{2}}-C_{O_{2}}\left(t_{0}\right)\right)-k_{L}\alpha \left(O_{2}\right)\times t$$

where $C^{*}{}_{O_{2}}$ and $C_{O_{2}}$ are the oxygen saturation concentration and oxygen concentration in the liquid, respectively. Assuming the liquid phase as homogenous and being $C_{O_{2}}\left(t_{0}\right)$ the oxygen concentration at *t* = 0, the volumetric mass transfer coefficient was determined by plotting $\ln\left(C^{*}{}_{O_{2}}-C_{O_{2}}\left(t_{0}\right)/C^{*}{}_{O_{2}}-C_{O_{2}}\left(t\right)\right)\;$ against time (*t*) [22,62].

Because the carbon source provided to the cultures as CO_2_ was used for the microalgae for their growth, the calculation of k_L_a(*CO*_2_) was estimated from the determination of the k_L_a(*O*_2_):

(2) $$k_{L}a\left(CO_{2}\right)=\sqrt{\frac{D_{CO_{2}}}{D_{O_{2}}}}\times k_{L}\alpha \left(O_{2}\right)$$

where $D_{CO_{2}}$ and $D_{O_{2}}$ are the diffusion coefficients of 1.99$\times 10^{-9}$ m^2^ s^−1^ and 2.41$\times 10^{-9}$ m^2^ s^−1^, respectively [22,62].

The k_L_a(CO_2_) was determined in the 20 L culture system. Subsequently, the k_L_a was determined in the plastic bags used in the 80 L culture systems, using three air flows to obtain a k_L_a value similar to the 20 L culture. With the above, it was evaluated whether, when maintaining the k_L_a in the 20 and 80 L cultures, although these had very different geometries, biomass production, phycoerythrin, carbohydrates, proteins, and lipids were conserved.

### 3.4. Biomass Analyses

#### 3.4.1. Cell Growth

Cell growth was followed by optical density (OD750nm) for 14 days using a spectrophotometer (Varioskan Flash, Thermo Fisher Scientific, Waltham, MA, USA). Growth was determined in triplicate in the 16 and 80 L cultures. Regarding the 400 L scale, its growth was determined in the five bags. Optical density was correlated with a previously performed standard dry weight curve. The standard dry weight curve was performed as described by Perez et al. [63] with slight modifications. Briefly, cells harvested by centrifugation (ST40R TX-750, Thermo Fisher Scientific, Waltham, MA, USA) at 4000 rpm for 15 min were washed twice with distilled water to eliminate salt residues. Then, algal cell suspensions were filtered through pre-weighed glass fiber filters (Whatman GF/F, 47 mm, nominal pore size 0.7 μm), and the filters were dried at 95 °C until constant weight, cooled down in a vacuum desiccator, and weighed again.

Biomass productivity (BP) was calculated according to:

(3) $$BP=\frac{DBW_{f}-DBW_{i}}{t}$$

where *BP* is the biomass productivity (mg L^−1^ d^−1^), *DBW_f_* is the final dry biomass weight (mg L^−1^), *DBW_i_* is the initial dry biomass weight in (mg L^−1^), and *t* is the cultivation time comprising final and initial measurement expressed in days [63].

The specific growth rate (*μ*) was calculated based on the following equation:

(4) $$\mu =\frac{lnX_{t}-lnX_{0}}{t-t_{0}}$$

where μ is the specific growth rate (d^−1^), *X_t_* represents the biomass concentration at the end of the exponential phase (g L^−1^), *X_0_* is the biomass concentration at the beginning, and *t − t_0_* corresponds to the duration of the exponential phase (d) [63].

Lastly, the doubling time (*DT*) expressed in days was determined, as shown in the following equation:

(5) $$DT=\frac{\ln\left(2\right)}{\mu_{max}}$$

where $\mu_{max}$ is the highest specific growth rate during the exponential phase of the culture [63].

#### 3.4.2. Total Carbohydrates Content

The analysis of carbohydrates was performed using a modified phenol-sulfuric method. Briefly, microalgae biomass was centrifuged at 4000 rpm for 15 min and washed twice with distilled water. The washed pellet was resuspended in distilled water. Then 200 μL of washed biomass was added to a glass tube, and 200 μL of a 5% aqueous phenol solution was added, followed by 1 mL of concentrated sulfuric acid. This mixture was incubated at 80 °C for 1 h and let sit to reach ambient temperature. The absorbance was measured at 488 nm in a spectrophotometer. A calibration curve was performed with glucose solutions in concentrations from 2 to 100 mg L^−1^ [64].

#### 3.4.3. Total Lipids and Fatty Acids Analysis

The total lipids of *P. purpureum* were measured according to the Bligh and Dyer method with modification [65]. Chloroform, methanol, and distilled water were added in ratios of 1:2:0.8 (*v*/*v*/*v*), respectively, into 100 mg of dried biomass. The resulting mixture was mixed for 30 s and then centrifuged at 2000 rpm for 10 min at 4 °C. After centrifugation, the lower layer was carefully recovered, filtered using a PTFE syringe filter (0.45 μm pore size, Thermo Fisher Scientific, Waltham, MA, USA), and transferred into a pre-weighed glass tube using a syringe. The remaining chloroform was dried in a concentrator at 45 °C, 2 h at 15 mm Hg. The extracted lipids were gravimetrically weighted to estimate the total lipid content, according to the following equation.

(6) $$Total\;lipid\;content=\frac{W-W_{0}}{DBW}\times \;100\%$$

where *W* is the weight of extraction vessel with oil expressed in g, *W*_0_ is the weight of empty extraction vessel in g, and *DBW* is the dry weight of algal biomass sample in g.

Fatty acids were determined through the derivatization of fatty acid methyl esters (FAMEs). Here, the sample obtained in the previous total lipid extraction method was suspended in 2 mL of hexane. This sample was mixed with 400 μL of internal standard (undecanoic acid 1000 mg/L in hexane/acetone 80:20 *v*/*v*) and 2 mL of methanol/sulfuric acid (93:7 *v*/*v*). This mixture was maintained for 60 min at 80 °C. Then, the sample was cooled to reach room temperature. After the addition of 5 mL of hexane, the sample was mixed in a vortex for 1 min, and two phases were formed. The organic phase was transferred to a volumetric flask (10 mL), and the re-extraction of the polar phase was carried out three to four more times until a volume of 10 mL was obtained. The FAMEs were analyzed with a Gas Chromatograph system (7820A, Agilent Technologies, Santa Clara, CA, USA), equipped with a SP2380 capillary column (30 m, 0.25 mm internal diameter, 0.20 μm film thickness) and flame ionization detector. Nitrogen was used as carrier gas at a flow rate of 0.8 mL min^−1^. The column temperature was kept initially at 50 °C for 2 min, followed by a ramp to reach 240 °C at a rate of 4 °C/min, and, at last, maintained at 240 °C for 1 min. An aliquot of the extract (1 μL) was injected automatically with 20:1 in split mode. Injector and detector temperatures were set at 260 and 280 °C, respectively [66].

#### 3.4.4. Total Protein Content

Protein extraction from *P. purpureum* was performed using 2 mL of culture media, centrifuged (4000 rpm, 15 min), and washed with distilled water. The obtained pellet was resuspended in 2 mL of NaOH 1M for 1 h at 100 °C. The extract obtained was centrifuged at 4000 rpm, 15 min, and 200 μL of the supernatant was taken to carry out the protein analysis described in the Modified Lowry Protein Assay Kit (Thermo Fisher Scientific, Waltham, MA, USA).

#### 3.4.5. Phycoerythrin Extraction and Purification

Phycoerythrin was extracted using a freeze–thaw method. A sample of 15 mL from *P. purpureum* culture was centrifuged at 4500 rpm for 15 min, and the supernatant was discarded. The pellet was mixed with 15 mL of distilled water and was vortexed until homogenization. The suspension was frozen at −20 °C for 2 h, and then it was thawed. The sample was vortexed until homogenization, and steps were repeated until completing four cycles. After that, the sample was centrifuged at 4500 rpm for 15 min. The pink supernatant was recovered and measured in a spectrophotometer at 564, 592, and 455 nm. The obtained data were substituted in the following equation to calculate the concentration of PE (mg mL^−1^) [55]:

(7) $$PE=\left[\left(OD_{564nm}-OD_{592nm}\right)-\left(OD_{455nm}-OD_{592nm}\right)\;0.2\right]\times 0.12$$

where OD is the optical density of the pigment at each wavelength, 0.2 and 0.12 are absorption coefficients for PE.

Furthermore, the phycoerythrin purity was evaluated by harvesting the cells and concentrated them using a membrane ultrafiltration unit designed and built by Membranology^®^ (Swansea, UK). This ultrafiltration unit was equipped with either 10,000 Molecular Weight Cut Off (MWCO) Daltons or 10 kDa membrane; these membranes allowed to obtain cells (retentate) and cell-free culture medium (permeate). The cell concentrate was subjected to the freeze–thaw method mentioned previously, and then the disrupted cells were processed in the ultrafiltration system with the 10 kDa membrane. In this case, the permeate contained phycoerythrin, and this extract was purified through a 2 kDa membrane. Finally, the purity index (PI) of the purified extract was estimated according to the following equation [67]:

(8) $$PI=\frac{OD_{564nm}}{OD_{280nm}}$$

where OD is the optical density of the pigment at each wavelength.

### 3.5. Statistical Analysis

Statistical analysis was performed using MINITAB 18^®^ software (Minitab, Inc., State College, PA, USA). One-way and multi-factor analyses of variance were used (ANOVA). Significant difference at *p* < 0.05 between the samples was determined by Tukey pairwise comparison. All experimental data were performed in triplicates (*n* = 3), and the obtained results are shown as ±SD (standard deviations), *n* = 3 in figures and tables.

## 4. Concluding Remarks

The scaling-up of *P. purpureum* culture was successfully conducted in an LED-illuminated hanging-bag photobioreactor, maintaining the volumetric mass transfer coefficient. The culture system presented is economical, easy to develop, and can be replicated to any desired volume. At the same time, it allowed obtaining high added-value compounds such as carbohydrates, lipids, proteins, and specific metabolites such as polyunsaturated fatty acids (palmitic, linoleic, arachidonic, and eicosapentaenoic acid) and phycoerythrin. The purification of the phycoerythrin was performed with membrane equipment that enabled processing large volumes of this pigment in a short period, facilitating the purification on a larger scale.

## Figures and Tables

**Table 1:** Volumetric mass transfer coefficient in the cultures with different airflows.

Volume (L)	Aeration (L min^−1^)	k_L_a (CO_2_) (s^−1^)
16	25	0.0052 ± 0.0005
80	50	0.0007 ± 0.0001
	70	0.0022 ± 0.0003
	80	0.0024 ± 0.0005

**Table 2:** Comparison of growth in the three different volume cultures.

Culture Volume (L)	Final Biomass Concentration (g L^−1^)	Biomass Productivity (mg L^−1^ d^−1^)	Specific Growth Rate µmax (d^−1^)	Doubling Time (d)
16 *	0.95 ± 0.052	68.64 ± 0.004	0.20 ± 0.011	3.45 ± 0.187
80 *	1.25 ± 0.081	92.25 ± 0.006	0.14 ± 0.008	4.85 ± 0.286
400 **	1.22 ± 0.049	85.42 ± 0.001	0.14 ± 0.002	5.10 ± 1.000

* ±±SD, *n* = 3; ** ±SD, *n* = 5.

**Table 3:** Total lipid content (as percentage of dry mass) and fatty acids profile (as percentage of total lipids) of *P. purpureum* cultivated in 16, 80 and 400 L.

Component	Lipids (%)		
	16 L	80 L	400 L
Total lipids	23.59 ± 2.77	15.16 ± 0.79	17.75 ± 2.58
(C8:0) Caprylic acid	3.05 ±1.30	11.83 ± 1.96	11.17± 1.85
(C16:0) Palmitic acid	40.41 ± 3.92	44.66 ± 4.12	46.03 ± 3.89
(C18:0) Stearic acid	2.28 ± 0.35	6.49 ± 0.71	6.25 ± 0.67
(C18:1n9c) Oleic acid	2.20 ± 0.11	4.25 ± 2.87	3.29 ± 2.70
(C18:2n6c) Linoleic acid	13.34 ± 0.60	7.93 ± 0.30	8.03 ± 0.29
(C20:4n6) Arachidonic acid	32.57 ± 5.11	22.34 ± 0.97	22.67 ± 0.91
(C20:5n3) Eicosapentaenoic acid	6.14 ± 0.28	2.50 ± 0.16	2.55 ± 0.15

±SD, *n* = 3.
